# The role of the mobile proton in fucose migration

**DOI:** 10.1007/s00216-019-01657-w

**Published:** 2019-03-02

**Authors:** Maike Lettow, Eike Mucha, Christian Manz, Daniel A. Thomas, Mateusz Marianski, Gerard Meijer, Gert von Helden, Kevin Pagel

**Affiliations:** 1 Department of Molecular Physics, Fritz Haber Institute of the Max Planck Society, Faradayweg 4-6, 14195 Berlin, Germany; 2 Institute of Chemistry and Biochemistry, Freie Universität Berlin, Takustraße 3, 14195 Berlin, Germany; 3 Present Address: Hunter College, The City University of New York, 695 Park Ave, New York, NY 10065 USA

**Keywords:** Fucose migration, Internal residue loss, Infrared spectroscopy, Mass spectrometry, Carbohydrates

## Abstract

Fucose migration reactions represent a substantial challenge in the analysis of fucosylated glycan structures by mass spectrometry. In addition to the well-established observation of transposed fucose residues in glycan-dissociation product ions, recent experiments show that the rearrangement can also occur in intact glycan ions. These results suggest a low-energy barrier for migration of the fucose residue and broaden the relevance of fucose migration to include other types of mass spectrometry experiments, including ion mobility-mass spectrometry and ion spectroscopy. In this work, we utilize cold-ion infrared spectroscopy to provide further insight into glycan scrambling in intact glycan ions. Our results show that the mobility of the proton is a prerequisite for the migration reaction. For the prototypical fucosylated glycans Lewis x and blood group antigen H-2, the formation of adduct ions or the addition of functional groups with variable proton affinity yields significant differences in the infrared spectra. These changes correlate well with the promotion or inhibition of fucose migration through the presence or absence of a mobile proton.

**Electronic supplementary material:**

The online version of this article (10.1007/s00216-019-01657-w) contains supplementary material, which is available to authorized users.

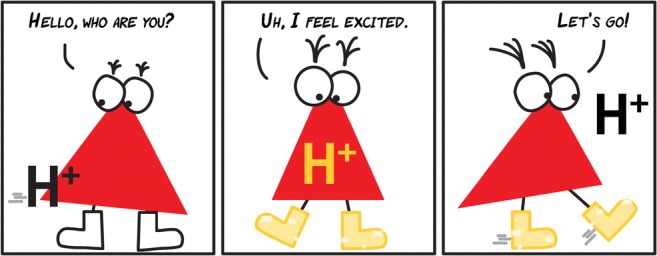

## Introduction

Glycans are among the most abundant biomacromolecules in living organisms and are essential in a variety of biological functions, such as cell-signaling and molecular recognition [1, 2]. The glycosylation patterns of proteins, lipids, or other organic molecules are in many cases highly dynamic, which is obvious in an evolutionary sense [3], but also accounts for physiological as well as pathological variations [4]. Fucose is a deoxy hexose found in a wide range of biologically relevant glycans and is easily distinguishable from many other hexoses due to the lack of the hydroxyl group at the C-6 position. In mammalian glycans, fucose is the fifth most abundant monosaccharide (7.2%), and nearly every fourth terminal end (23.8%) carries a fucose [5]. Among human milk oligosaccharides, up to 80% are fucosylated, depending on the individual’s secretor status [6]. Glycans attached to proteins are usually fucosylated in the Golgi apparatus or in the endoplasmic reticulum during the final step of glycan processing by different fucosyltransferases [7]. Altered fucosylation can account for pathological variations, for example, the overexpression of fucose in cancer cells. The fucosylated Lewis y (Le^y^) antigen is highly expressed on a wide range of tumors, and tumor growth can be inhibited by suppressing Le^y^ expression [4, 8, 9].

The inherent structural diversity of glycans and the limited availability of standards have made glycan structural characterization a major analytical challenge. Tandem mass spectrometry (MS) is a powerful tool in the analysis of glycan structures [10–13] and is often combined with chromatography [14] and/or ion mobility spectrometry [15, 16]. In general, tandem MS can provide the mass of the intact ion and, additionally, the masses of its fragment ions in a single experiment. Different types of fragmentation techniques yield distinct types of fragment ions that provide information on the original glycan sequence. Information-rich fragment spectra contain glycosidic fragments and/or cross-ring fragments, but can also show rearrangement products and internal fragments. An effective application of MS in glycan analysis therefore requires comprehensive knowledge of potential gas-phase reactions and fragmentation pathways.

Suitable techniques to shed light on the mechanism of a gas-phase reaction in mass spectrometry are infrared (IR) spectroscopy [17, 18], ion mobility-mass spectrometry (IM-MS) [19, 20], hydrogen-deuterium exchange (HDX) [21], and stable isotope labeling [22] or computational modeling [23]. A very prominent rearrangement reaction in biomolecules that has been investigated extensively in the last years is peptide scrambling. Peptide scrambling in protonated or multiply protonated *b*-type peptide ions involves a head-to-tail macrocyclization and subsequent reopening reaction prior to fragmentation in tandem MS with collision-induced dissociation (CID). Consequently, the original sequence information is lost during scrambling, which may result in erroneous assignments. The phenomenon was first reported in 1997 [24]. Since the beginning of the 2000s, the underlying mechanism and the structure of the macrocyclic intermediate have been successfully proven using mainly a combination of IR spectroscopy and computational modeling [25]. It has been found that the incidence of this gas-phase rearrangement is negligible in shotgun proteomics but of major interest in understanding the fragmentation pathways in CID [26–28].

For more than 20 years, researchers have also been studying glycan rearrangement reactions in tandem MS experiments [29, 30]. Most of these studies are based on the observation of unusual fragment masses that arise from a rearrangement of fucose and occasionally other monosaccharide units such as xylose [31] during the CID process. This phenomenon is also often referred to as internal residue loss (IRL) and has been studied extensively by probing the influence of different adduct ions [32, 33] or an aglycon [34] and different derivatization strategies [35–37]. Based on these findings, various mechanisms have been proposed [30, 34], all of which involve a mobile proton that is able to migrate to the reactive site. However, despite these efforts, the underlying mechanism is not fully understood to date.

**Fig. 1:**
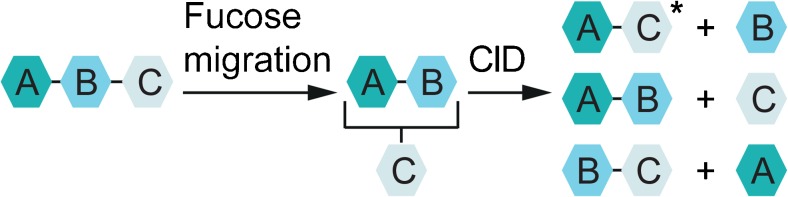
Glycan rearrangement reactions consist of two independent, consecutive steps: fucose migration and then collision-induced dissociation (CID) with internal residue loss (IRL) products and glycosidic fragments. The energy barrier for a fucose migration is low and the product of a fucose migration reaction is stable. The IRL fragment is labeled with an asterisk. A, B, and C represent monosaccharides. Glycosidic linkages are indicated with bars

Recently, we reported a glycan sequence scrambling reaction in the intact trisaccharide ions Lewis x (Le^x^) and blood group antigen H-2 (BG-H2), which are substructures of the tetrasaccharide Lewis y (Le^y^) [38]. The observed fucose migration involves the cleavage of a glycosidic bond and the subsequent or concerted formation of a new glycosidic bond (Fig. 1). The reaction takes place in the presence of a proton and is inhibited in its absence, namely in the sodium adducts. Interestingly, this reaction was found to occur under soft conditions without substantial collisional activation, which indicates a rather low-energy barrier for the rearrangement. Based on these results, we concluded that fucose residues could spontaneously migrate from the terminal end of a glycan chain to adjacent or remote intramolecular sites of the glycan without CID. The previously reported occurrence of unexpected fragment masses in IRL is therefore likely only a result, but not the cause of fucose migration.

Here, we build on our previous findings and investigate the role of the mobile proton in fucose migration in the intact ion pair BG-H2 and Le^x^ using cold-ion IR spectroscopy. In order to increase or diminish the mobility of protons in the investigated ions, functional groups and adduct ions with competitive proton affinity have been utilized. Our results clearly show that a mobile proton at a specific site of the molecule is required for fucose migration.

## Experimental

### Sample preparation

Commercially available Lewis and blood group antigens were purchased from Dextra Laboratories (Reading, UK) and Biozol (Eching, DE) and used without further purification. Solvents (HPLC grade) and labeling reagents were purchased from Sigma-Aldrich (St Louis, USA) and used without further purification. Aqueous glycan stock solutions (1 mM and 100 μM for labeled glycans) were further diluted prior use with water/methanol (*v/v*, 50/50) to yield 20–500 μM analyte solutions. To promote the adduct formation in the measurements of [M+NH_4_]^+^, [M+NMe_3_H]^+^ and [M+NEt_3_H]^+^, ammonium acetate, trimethylammonium chloride, and triethylammonium acetate, respectively, were added in concentrations of 1–5 mM.

### Synthesis of labeled glycans

For glycan labeling, the Lewis and blood group antigens were dissolved in water to yield 100 mM stock solutions. The stock solutions were divided into 10 μL (1 μmol) aliquots and freeze-dried. The glycans were labeled with 2-aminobenzoic acid (AA) and 4-amino-*N*-(2-diethylaminoethyl) benzamide (procainamide) via reductive amination using standard protocols [39]. Removal of the excess label was performed using paper chromatography [40]. The labeled glycans were further purified using HyperSep Hypercarb SPE cartridges (ThermoFisher Scientific, Waltham, Massachusetts, US) according to manufacturer’s instructions. The labeled glycans were then freeze-dried and dissolved to aqueous 100 μM stock solutions.

### Cold-ion IR spectroscopy

**Fig. 2:**
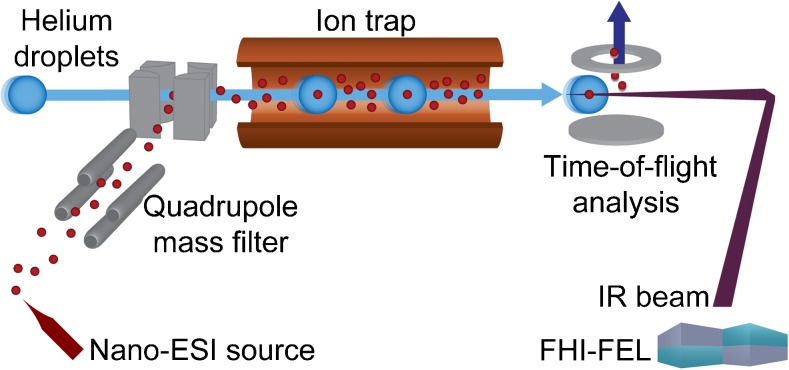
Schematic view of the experimental setup and the attached Fritz Haber Institute free-electron laser (FHI-FEL). The setup consists of a nano-electrospray ionization (nano-ESI) source, a quadrupole mass filter and a helium-droplet source, which is on-axis with a coolable ion trap and the IR laser beam

The experimental setup (Fig. 2) has been comprehensively described and the interested reader is referred to the literature [41]. Briefly, glycans are ionized by nano-electrospray ionization, mass-to-charge selected in a quadrupole mass filter and stored in a hexapole ion trap. The ions are picked up by traversing superfluid helium nanodroplets and rapidly cooled to the equilibrium temperature of the superfluid helium bath (0.37 K). The helium droplets, optically transparent within the IR range, show only weak interactions with the embedded molecular ions and therefore do not contribute to the optical spectra. In the detection region, the embedded ions are irradiated with IR light provided by the Fritz Haber Institute IR free-electron laser (FHI-FEL). The FEL macropulse (duration of 10–15 μs) comprises micropulses with a duration of ca. 10 ps separated by 1 ns (1-GHz micropulse repetition rate). Following the absorption of a resonant photon within a micropulse, the ions are cooled to the vibrational ground state via helium evaporation from the droplet. After the absorption of multiple photons from discrete micropulses, the ion is ejected from the droplet and detected in a time-of-flight mass analyzer. The IR spectrum is recorded as the integrated ion intensity as a function of the laser wavelength in the fingerprint range at the maximum from 900 to 1800 cm^−1^. For each molecule, at least two independent scans are measured and averaged.

## Results and discussion

### Ammonium adduct ions

**Fig. 3:**
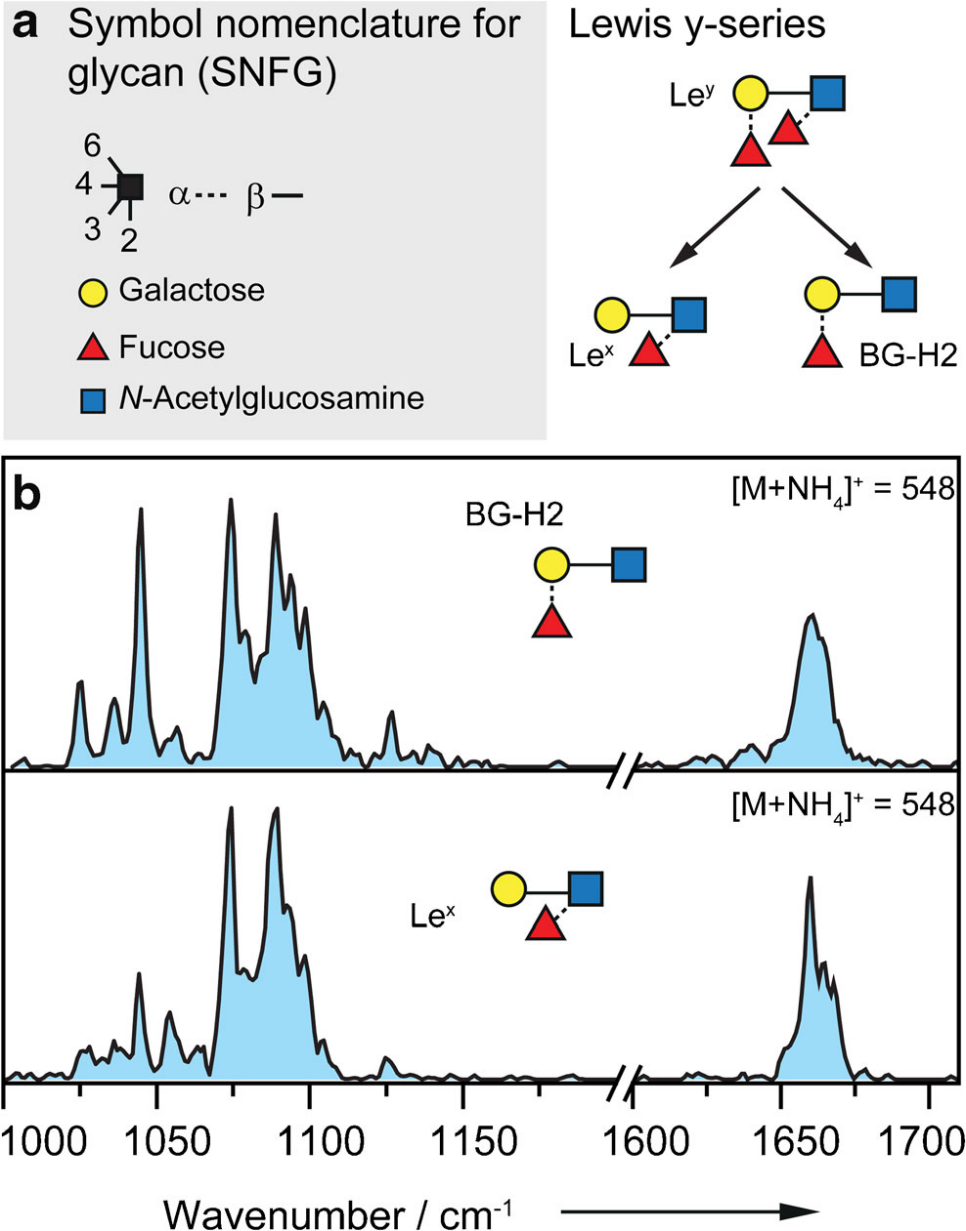
**a** Symbol nomenclature for glycan (SNFG, left panel) [42] and the Lewis y series (right panel) with the tetrasaccharide Le^y^ and the two trisaccharides BG-H2 and Le^x^ that are substructures of Le^y^. **b** IR spectra of the parent ions BG-H2 (upper panel) and Le^x^ (lower panel) investigated as [M+NH_4_]^+^ species (*m/z* 547) in the range of 1000–1200 cm^−1^ and 1600–1710 cm^−1^. The two IR spectra are nearly identical

First, trisaccharides of the Lewis y-series (Le^y^-series) were investigated as ammonium adducts [M+NH_4_]^+^ (*m/z* 547). The Le^y^-series consists of the tetrasaccharide Le^y^ and the two trisaccharides BG-H2 and Le^x^ that are substructures of Le^y^ (Fig. 3a; Electronic Supplementary Material (ESM) Fig. S1). The IR spectra of the trisaccharide standards BG-H2 and Le^x^ are shown in Fig. 3b and were recorded using the instrumental setup described above. The instrument settings were tuned to minimize activation of the parent ion. As expected, both IR spectra show well-resolved features in the range of 1000 cm^−1^ to 1700 cm^−1^. Generally, vibrations in glycans around 1100 cm^−1^ are associated with numerous C–O and coupled C–O–C–O-stretch vibrations of the (hemi-)acetal. Features above 1500 cm^−1^ and around 1650 cm^−1^ canonically stem from the amide II (N–H bend) and amide I (C=O stretch) vibrations in *N-*acetylglucosamine monosaccharides. Weaker vibrations between 1200 and 1500 cm^−1^ are O–H-bending modes. With a charged site at the amide group, the amide vibrational modes can be strongly shifted, e.g. with a proton at the oxygen, the amide I vibration is likely red-shifted. A confident assignment and differentiation of the amide I and II features can therefore not be made.

The IR spectrum of BG-H2 as [M + NH_4_]^+^ (Fig. 3b, upper panel) shows nine well-resolved bands in the range from 1000 to 1200 cm^−1^. The strongest absorptions are found at 1045, 1075 and 1089 cm^−1^. Between 1200 and 1600 cm^−1^, weaker absorption bands, which are not resolvable with the utilized instrumental setup, are observed. A broad absorption is found in the higher wavenumber range of the spectrum around 1660 cm^−1^. A comparison of the IR spectrum of BG-H2 with that of Le^x^ as [M+NH_4_]^+^ ion (Fig. 3b, lower panel) reveals many similarities. The main absorptions in the IR spectrum of Le^x^ are found between 1000 and 1200 cm^−1^ and around 1660 cm^−1^. In the lower wavenumber range, the strongest absorptions are at 1075 and 1089 cm^−1^ and in the higher wavenumber range, a broad absorption is found around 1660 cm^−1^.

In general, all features in the IR spectrum of Le^x^ are also present in the spectrum of BG-H2, however, with varying intensity, which likely results from differing ratios of alpha and beta anomers in the gas phase. The nearly identical IR spectra of the intact ammonium adduct ions therefore imply that the trisaccharides BG-H2 and Le^x^ rearrange to the same chemical structure, not only as protonated ions as observed previously [38] but also as ammonium adduct ions. This is in good agreement with previously reported CID experiments in which IRL reactions have been found for ammonium adducts [33].

**Fig. 4:**
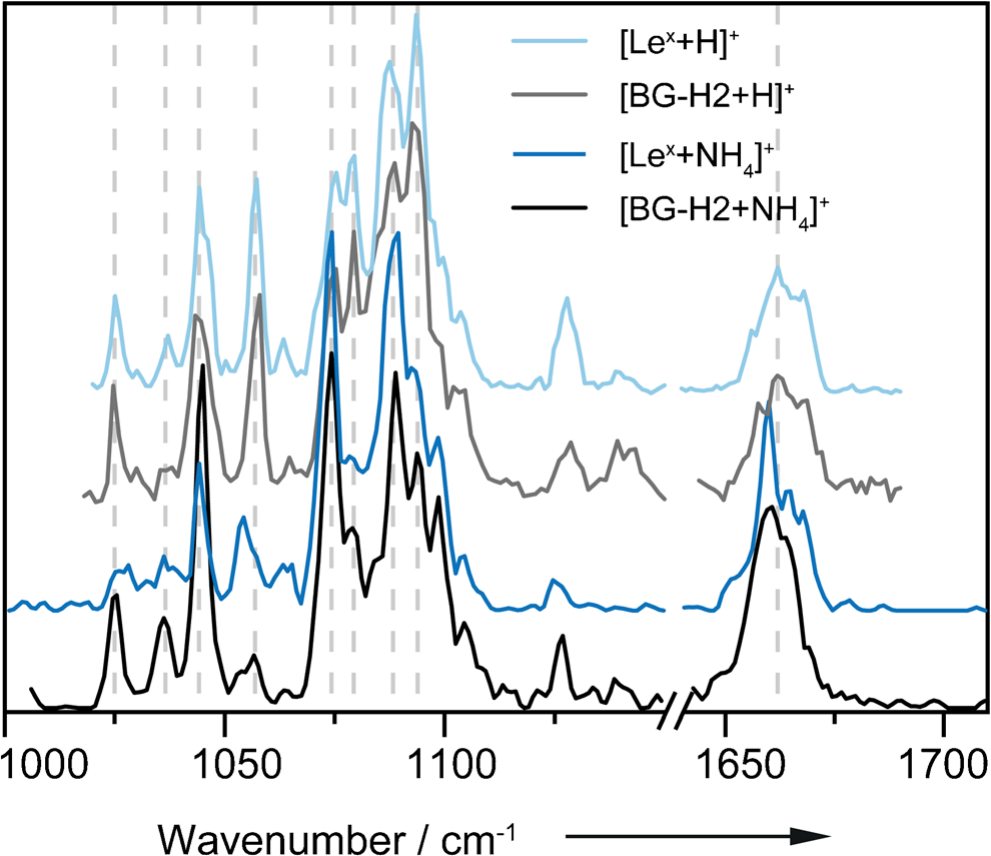
Stacked overlay of the IR spectra of the parent ions BG-H2 and Le^x^ investigated as [M+H]^+^ and [M+NH_4_]^+^ species in the range of 1000–1710 cm^−1^ (break between 1150 and 1640 cm^−1^). The spectra of the protonated species are reproduced from Mucha et al. [38] and shown in the of 1020–1690 cm^−1^. The four IR spectra are highly similar

Observing fucose migration in protonated ions and ammonium adducts at almost identical instrument conditions indicates a comparable energy barrier, migration mechanism and possibly even structure in the two types of ions. In order to test this hypothesis, the optical signatures of protonated and ammonium adduct ions of the Le^y^ series are compared. Remarkably, very similar IR signatures are observed for the isomers BG-H2 and Le^x^, regardless whether they are present as [M+NH_4_]^+^ or [M+H]^+^ species. The stacked overlay in Fig. 4 reveals that the main features are qualitatively present in all four IR spectra (indicated by vertical dashed lines). This is a rather surprising observation, as different adduct ions typically change the gas-phase conformation of the molecule substantially and therefore lead to distinct IR traces. A possible explanation for the striking similarities observed here is that the present species are in fact not strict [M+NH_4_]^+^ adducts, but rather neutral ammonia adducts of a protonated glycan [NH_3_+MH]^+^. The proton directly transfers to the glycan while the ammonia is coordinated as the neutral species, which does not seem to change the gas-phase conformation of the glycan. With only weak interactions of the neutral ammonia molecule, the observed absorptions largely correspond to those of the protonated glycan ion, which in turn is able to undergo fucose migration.

Ammonia loss can be an indicator for the [NH_3_+MH]^+^ species. Recorded as [M+H]^+^ ions, the ammonia loss can either be produced by varying the cone voltage or also after ejection from the helium nanodroplet. The latter has been recorded over all scans. A fraction of [M+H]^+^ ions are detectable in each scan indicating loosely bound ammonia. It should be pointed out that the [M+H]^+^ ion signal is recorded with each scan but it is not part of the resulting IR spectrum of the [M+NH_4_]^+^ species.

Gas-phase proton transfer is one of the most commonly observed processes in ion-molecule chemistry and studies have provided a substantial database of thermochemical parameters. For singly charged species, the protonation propensity is predominantly governed by the gas-phase proton affinity (PA) of the present functional groups. The gas-phase basicity (GB) and the PA are directly derived from the reaction of a neutral molecule with a proton to form the protonated species, with the GB corresponding to the Gibbs free energy change and the PA as the respective enthalpy change [43]. In the trisaccharides BG-H2 and Le^x^, the proton is likely located at the C=O of the amide (PA 230 kcal mol^−1^ for C=O compared to 220 kcal mol^−1^ for the N–H group) [44]. Even though the actual proton affinity can deviate depending on the local environment, the PA of free ammonia is considerably lower (204 kcal mol^−1^) [43] which supports the formation of a [NH_3_+MH]^+^-type ion and the presence of a mobile proton.

### Alkylammonium adduct ions

**Fig. 5:**
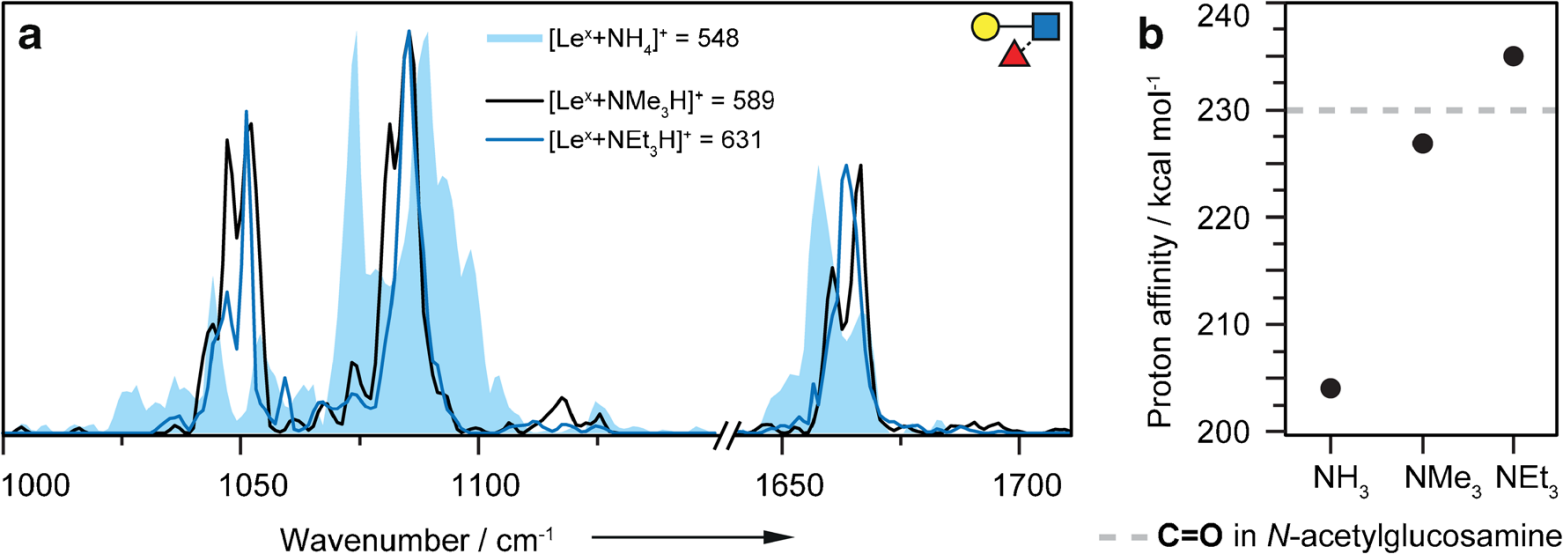
**a** Overlay of the IR spectra of [Le^x^+NMe_3_H]^+^ (black line), [Le^x^+NEt_3_H]^+^ (blue line) and [Le^x^+NH_4_]^+^ (blue filled) in the range of 1000–1710 cm^−1^ (break between 1150 and 1640 cm^−1^). The spectra of the alkylammonium adducts are very similar and differ from that of the ammonium adduct of Le^x^. **b** Proton affinity of different adduct ions in kcal mol^−1^ [43] compared to the proton affinity of the C=O group in a representative *N*-acetylglucosamine (gray line) [44]. Due to the local environment, the PA of the C=O group in Le^x^ can be slightly different

To further test the impact of proton mobility, the trisaccharide Le^x^ was investigated with two alkylammonium adduct ions as [Le^x^+NMe_3_H]^+^ and [Le^x^+NEt_3_H]^+^ (Fig. 5). The spectra of BG-H2 with different alkylammonium adduct ions could not be recorded as a result of unstable electrospray conditions. As shown in Fig. 5b, trimethylammonia (PA 227 kcal mol^−1^) and triethylammonia (PA 235 kcal mol^−1^) [43] have substantially higher gas-phase PAs than ammonia. A proton transfer from the alkylammonium ions to the glycan as observed for ammonium ions is therefore energetically unlikely. The spectra of [Le^x^ + NMe_3_H]^+^ and [Le^x^ + NEt_3_H]^+^ shown in Fig. 5a confirm this hypothesis. The two traces are almost identical, but differ considerably from the spectrum of [Le^x^+NH_4_]^+^. The spectra of the alkylammonium adducts show two well-resolved bands at 1050 cm^−1^ and 1085 cm^−1^ of which the first is a split band. In the higher wavenumber range, a peak is found, which in comparison to the ammonium adduct is slightly blue-shifted. The only noticeable difference in the two alkylammonium adduct spectra are a side peak at 1081 cm^−1^ in the spectrum of [Le^x^+NMe_3_H]^+^ and a subtle shift of the feature at higher wavenumbers. However, the tremendous difference to the spectrum of the ammonium adduct is apparent. Alkylammonium adduct ions demobilize the proton or at least offer two possible protonation sites. Therefore, the gas-phase structures of the alkylammonium adducts of Le^x^ are rather similar while those of the ammonium adduct closely resembles that of the protonated species.

### Fluorescently labeled glycan ions

**Fig. 6:**
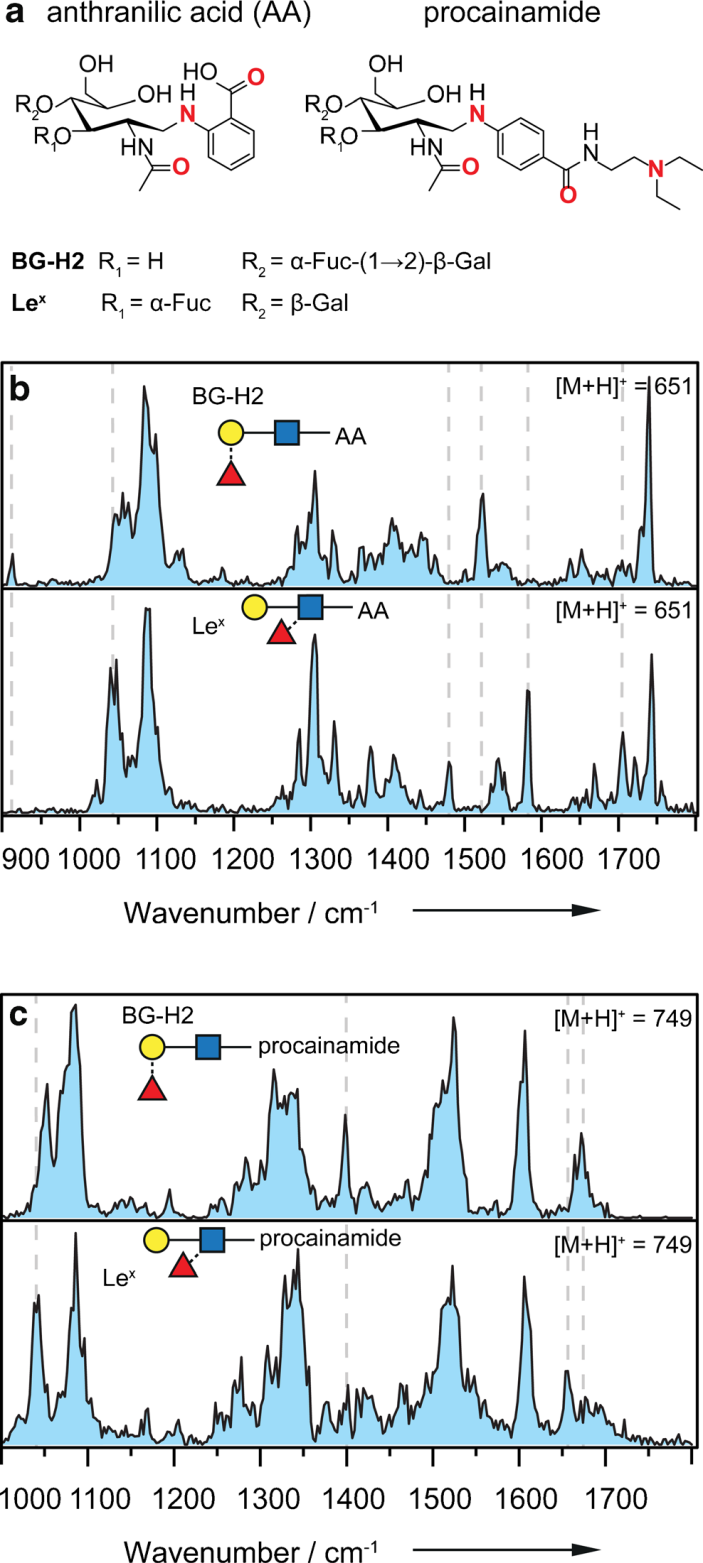
**a** Chemical structures of the investigated, labeled trisaccharides. Bold red atoms indicate possible protonation sites. **b** IR spectra of the parent ions Le^x^-AA (upper panel) and BG-H2-AA (lower panel) investigated as [M+H]^+^ species (*m/z* 651) in the range of 900–1800 cm^−1^. The two IR spectra are distinguishable from each other, and the gray dashed lines indicate major differences. **c** IR spectra of Le^x^-procainamide (upper panel) and BG-H2-procainamide (lower panel) investigated as [M+H]^+^ species (*m/z* 749) in the range of 1000–1800 cm^−1^. For both isomeric sets, the two IR spectra are clearly distinguishable from each other, and the gray dashed lines indicate major differences

Glycans are challenging to study with common analytical techniques, especially at low analyte concentrations. A modification of the sugars with fluorescent labels facilitates their analysis and quantification by liquid chromatography using fluorescence detection. Since glycans themselves do not fluoresce, they are typically modified with a fluorophore at their reducing end by reductive amination. Popular chromophores in LC are 2-aminobenzamide (2-AB), anthranilic acid (AA) or 4-amino-*N*-(2-diethylaminoethyl) benzamide (procainamide) [45, 46]. Labeling often goes in-line with improved protonation efficiency due to the presence of one or more apparent basic sites at the label. As a result, fluorescence labeling is also beneficial for hyphenated LC-MS techniques [47]. However, to date relatively little is known about the impact of fluorescence labeling on the previously described fucose migration reaction. In order to evaluate this, the trisaccharides of the Le^y^-series were modified with the common AA (*m/z* 651) and procainamide-labels (*m/z* 749) (Fig. 6a). IR spectra of the protonated ions [M+H]^+^ were recorded in the range from 900 to 1800 cm^−1^ and 1000 to 1800 cm^−1^, respectively, and the obtained traces are shown in Fig. 6b and c.

In the range from 1000 to 1200 cm^−1^, the dominant vibrations arise from the typical C–O and coupled C–O–C–O-stretch vibrations of the (hemi-)acetal. In stark contrast to the spectra of the unmodified trisaccharides, all four spectra show a large number of vibrations in the range from 1250 to 1500 cm^−1^, which are predominantly associated with functional groups of the labels. In the last part of the spectra, from 1500 to 1800 cm^−1^, the vibrations derive either from the amide vibrations in the *N-*acetylglucosamine or from the label.

In AA-labeled glycans (Fig. 6b), vibrations above 1700 cm^−1^, stem from the C=O stretch in the carboxylic acid at the aromatic ring. The IR spectra of the AA-labeled glycans show well-resolved bands but also broad features. The latter may result from a larger set of accessed low-energy conformers and/or protonation isomers (protomers) [48]. In the IR spectrum BG-H2-AA, the main features are found at 1087, 1305, 1523, and 1740 cm^−1^. In the IR spectrum of the isomeric trisaccharide Le^x^-AA, the main absorptions are at 1044 and 1087 cm^−1^ in the lower wavenumber range and at 1305 and 1582 cm^−1^ and at 1705 and 1743 cm^−1^ in the higher wavenumber range. The IR fingerprints of the two isomeric trisaccharides differ. Especially above 1450 cm^−1^, the vibration bands are diagnostic. The main differences in the IR spectra are marked with vertical gray dashed lines.

In procainamide-labeled glycans (Fig. 6c), a second amide group and a tertiary amine contribute to the IR spectrum. The spectra of BG-H2-procainamide and Le^x^-procainamide show less well-resolved features than the spectra of the AA-labeled derivatives. In both spectra, strong amide vibrations are found from 1500 to 1700 cm^−1^. The spectral signatures or fingerprints of the two isomeric trisaccharides are clearly distinguishable from each other.

Based on the observed spectra, both labels—anthranilic acid and procainamide—seem to alter the proton mobility in the Le^y^ series, which leads to an inhibition of fucose migration. Based on the data, it is not possible to exclude a rearrangement reaction; however, the observed chemical structures are clearly different.

For procainamide, these results are consistent with previous CID experiments, which show that this label reduces or inhibits IRL reactions in fucosylated glycans [49]. Glycans labeled with anthranilic acid, on the other hand, have been reported to produce unknown mass-to-charge signals from IRL reactions in tandem MS experiments [50]. A possible explanation for this different behavior might be the competition between protonation sites. In AA-labeled glycans, the proton has at least two possible protonation sites with comparable affinity: the amide group on the *N*-acteylglucosamine and the secondary, aromatic amine. The former would facilitate a migration reaction, while the latter would inhibit it. In the procainamide label, the basic tail with a tertiary amine probably has the highest proton affinity while the secondary, aromatic amine and the aromatic amide have compatible affinities to the *N*-acteylglucosamine. It is therefore conceivable that in both cases more than one protomer is populated at room temperature, a migration, however, is—if at all—only conceivable in case of the AA label. It can therefore be concluded that an additional functional group with comparable or higher proton affinity inhibits or at least alters the fucose migration reaction in the isomeric trisaccharides of the Le^y^-series.

## Conclusion

Here, we use cold-ion IR spectroscopy to study the impact of adduct formation and proton mobility on fucose migration reactions in the glycan isomers BG-H2 and Le^x^. In ammonium adducts, the proton is located at the glycan in the form of a [NH_3_+MH]^+^-type ion of the individual isomers. These ions are in many ways similar to [MH]^+^ ions and show fucose migration to the same, but yet not fully unraveled, chemical structure. In the alkylammonium adducts of Le^x^, on the other hand, the proton is immobilized at the adduct, which leads to a distinct glycan structure in which fucose migration is likely not occurring. A similar behavior was observed when different functional groups with competitive proton affinity are introduced. In these structures, fucose migration to one chemical structure is inhibited or at least altered, since multiple competing protonation sites in the glycan reduce proton mobility. These results agree well with our previous results in which fucose migration is inhibited in the sodium adducts.

In summary, our results suggest that a proton needs to be localized on the trisaccharide and presumably in close proximity to the rearranging monosaccharide for the fucose migration reaction to take place. This observation validates what has already been found in work on IRL. The proton mobility is influenced in the presence of adduct ions or functional groups with competitive proton affinity.

## Electronic supplementary material

ESM 1 (PDF 168 kb)
