# Supplementary material for: The role of the mobile proton in fucose migration
Source: Anal Bioanal Chem. 2019 Mar 2;411(19):4637–45. doi: 10.1007/s00216-019-01657-w (PMC6611747; doi:10.1007/s00216-019-01657-w)
Supplement: Supplementary file 1 — (PDF 168 kb) [file 216_2019_1657_MOESM1_ESM.pdf]

Analytical and Bioanalytical Chemistry

Electronic Supplementary Material

The role of the mobile proton in fucose migration

Maike Lettow, Eike Mucha, Christian Manz, Daniel A. Thomas, Mateusz Marianski, Gerard Meijer, Gert von Helden, Kevin Pagel

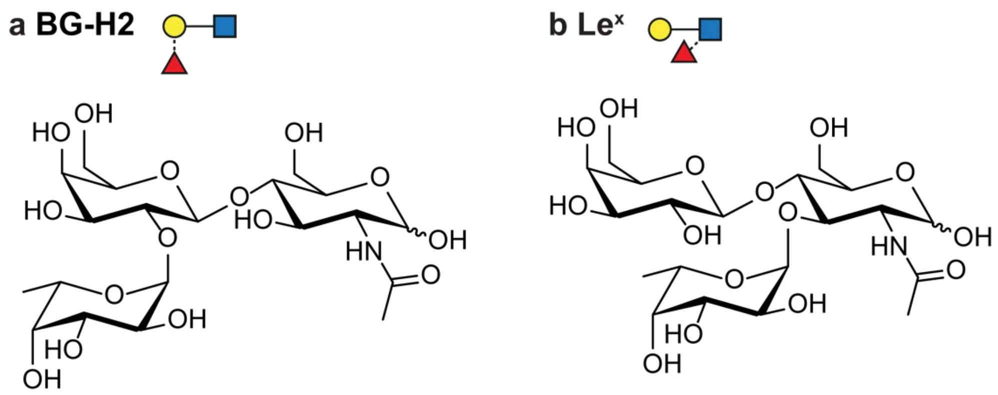

Fig. S1 Chemical structures and SNFG representation of the investigated trisaccharides **a** Lewis x (Le^{x}) and **b** blood group H-2 (BG-H2)
